# A Metacommunity Framework for Enhancing the Effectiveness of Biological Monitoring Strategies

**DOI:** 10.1371/journal.pone.0043626

**Published:** 2012-08-24

**Authors:** Tadeu Siqueira, Luis M. Bini, Fabio O. Roque, Karl Cottenie

**Affiliations:** 1 Departamento de Ecologia, Universidade Estadual Paulista – UNESP, Rio Claro, São Paulo, Brazil; 2 Departamento de Ecologia, Universidade Federal de Goiás, Goiânia, Goiás, Brazil; 3 Departamento de Biologia, Universidade Federal de Mato Grosso do Sul, Campo Grande, Mato Grosso do Sul, Brazil; 4 Department of Integrative Biology, University of Guelph, Guelph, Ontario, Canada; University of San Diego, United States of America

## Abstract

Because of inadequate knowledge and funding, the use of biodiversity indicators is often suggested as a way to support management decisions. Consequently, many studies have analyzed the performance of certain groups as indicator taxa. However, in addition to knowing whether certain groups can adequately represent the biodiversity as a whole, we must also know whether they show similar responses to the main structuring processes affecting biodiversity. Here we present an application of the metacommunity framework for evaluating the effectiveness of biodiversity indicators. Although the metacommunity framework has contributed to a better understanding of biodiversity patterns, there is still limited discussion about its implications for conservation and biomonitoring. We evaluated the effectiveness of indicator taxa in representing spatial variation in macroinvertebrate community composition in Atlantic Forest streams, and the processes that drive this variation. We focused on analyzing whether some groups conform to environmental processes and other groups are more influenced by spatial processes, and on how this can help in deciding which indicator group or groups should be used. We showed that a relatively small subset of taxa from the metacommunity would represent 80% of the variation in community composition shown by the entire metacommunity. Moreover, this subset does not have to be composed of predetermined taxonomic groups, but rather can be defined based on random subsets. We also found that some random subsets composed of a small number of genera performed better in responding to major environmental gradients. There were also random subsets that seemed to be affected by spatial processes, which could indicate important historical processes. We were able to integrate in the same theoretical and practical framework, the selection of biodiversity surrogates, indicators of environmental conditions, and more importantly, an explicit integration of environmental and spatial processes into the selection approach.

## Introduction

Planning for biodiversity monitoring and conservation strategies is challenging, not only because biodiversity is threatened by multiple factors (e.g., habitat fragmentation, climate change, and invasive species [1]), but also because biodiversity itself is maintained by multiple factors [2]. Therefore, conservation strategies should ideally be based on information derived from varying levels of complexity. However, due to the paucity of funds, time, and knowledge, and because it is not possible to survey the distribution of all organisms, the use of biodiversity indicators and surrogates is often suggested as a way to reconcile these opposing forces of complexity and practicality [3].

The use of biological indicators is essential in tropical regions, where estimates of species richness are uncertain. Also, these regions are plagued by the lack of knowledge of species’ identity and geographical distribution, the so-called Linnean and Wallacean shortfalls, respectively [4], [5]. The rationale for using indicators is to reduce the complexity associated with biodiversity into practical, less costly, and more quickly obtainable measures that can be used for biodiversity conservation and monitoring. This approach is mainly based on two key assumptions: (i) that an indicator group represents a major component of the entire biodiversity of an area [6], and (ii) that an indicator responds to the same ecological processes that generate and maintain overall biodiversity [7]. To date, most studies on the performance of surrogacy approaches have addressed the first assumption, and have analyzed the effectiveness of using the species richness of certain groups as indicators of overall biological diversity and environmental changes [8]. Within these groups, several have been regarded as good indicators, including butterflies, some aquatic insects, birds, and primates [9]–[11]. However, indicator-species richness is not informative about patterns of community composition within and between assemblages [12]. Thus, there has been a shift toward the use of multiple indicators [13], complementarity-based analyses [14], and more recently, multivariate methods aiming to measure patterns of community concordance among different taxonomic groups [15], [16]. Community concordance (or congruence) exists when two or more groups of taxa exhibit similar spatial patterns of variation in community structure. When a strong pattern of congruence is found, one can use a given group as a surrogate for others. The resources saved could then, for example, be used to increase the spatial coverage of a biodiversity inventory program [17].

One fundamental problem of all these surrogacy approaches is the lack of information on whether indicator taxa respond similarly to the main processes that structure overall biodiversity. A recent synthesis suggested the use of the metacommunity framework to study these structuring processes [18]. A metacommunity consists of groups of local communities that are linked by dispersal of multiple, potentially interacting species, and are structured by both environmental and spatial processes [19]. Metacommunity theory is organized in the following four frameworks, depending on the relative influence of these processes on community structure: species sorting, patch dynamics, neutral model, and mass effects [18]. These frameworks, however, may represent processes that act simultaneously in some communities, and cannot be viewed independently of each other, but rather as points along a continuum [20]. At one extreme, it is assumed that individuals are identical in their fitness, and that variation in community composition is driven mainly by stochastic processes (the neutral model [21]); and at the other extreme, variation in the metacommunity is determined by the responses of different species to environmental gradients. The other two frameworks can be seen as special cases of the species sorting framework [22]. In patch dynamics, the interacting species differ from each other in their abilities as either good competitors or good colonizers within a uniform environment [18]. Within a heterogeneous environment, strong priority effects caused by dispersal limitation can lead to different and stable communities. For the mass effects framework, intensive dispersal allows species to exist at sites that are normally considered marginal or outside of their environmental range [18]. Following this reasoning, one could hypothesize that some communities are composed of groups of species that conform to environmental processes, and others are more influenced by spatial processes (e.g., [23], [24]). Despite the recent interest in empirical tests of metacommunity theory (see review by Logue [25]), there is still unexploited potential for the metacommunity approach to inform conservation approaches [26]. We argue that beyond analyzing whether certain taxonomic groups can be used as indicators of overall biological diversity, we need to know whether indicator taxa also show similar responses to the main structuring processes affecting the entire metacommunity.

In this study, we evaluated the effectiveness of indicator taxa in representing spatial variation in the macroinvertebrate community composition in Atlantic Forest streams, and the processes that drive this variation. We focused on analyzing whether some groups respond better to environmental factors and others are more influenced by spatial processes, and on how this can help in deciding which indicator taxa should be used in biomonitoring programs. We specifically investigated (i) whether indicator taxa are good surrogates of the variation in community composition of entire metacommunities. More importantly, we also investigated (ii) whether indicator taxa respond congruently to structuring processes affecting the entire metacommunity, and (iii) whether the performance of an indicator taxon depends on its identity or on the amount that it contributes to the completeness of the dataset. This is worthwhile because previous studies suggested that, after controlling for the effect of species richness, random subsets of species may perform better than indicator taxa [27]. Therefore, comparing the performance of predetermined indicators against random subsets of taxa in representing biological diversity is a necessary step toward the acceptance of their effectiveness [28]. Finally, if random subsets perform better than classical indicator taxa, we would be able to (iv) define potential indicator groups by choosing those subsets that best respond to environmental gradients.

## Methods

### Study Area and Dataset Analyzed

The dataset that we used was extracted from the “Macroinvertebrate database” compiled by the research group in aquatic entomology of the Universidade Federal de São Carlos, Brazil (see details in [11], [29], [30]). Thirty-nine sites located in the Atlantic Forest (state of São Paulo; see [31] for a discussion on the ecological importance and high level of threat of this biome) were selected. Of these, 20 were located in protected areas and 19 in areas fragmented by agricultural activities.

This dataset includes information on abundances of genera, together with local and landscape environmental variables. Sampling and measured environmental variables are detailed elsewhere [11]. Although several studies have focused on how local and landscape environmental factors influence the distribution and abundance of macroinvertebrates in streams [11], [32], there is no consensus about which scales and factors are the most influential, especially for tropical streams [30], [33]. Therefore, we included predictors from different scales to increase the probability that at least some variables might account for different species’ niche requirements. Within each scale (i.e., local, landscape or regional), these variables are considered important determinants of aquatic macroinvertebrate distribution in streams [34], [35]. Examples include physical (water temperature, stream depth and width) and chemical (pH, dissolved oxygen, electrical conductivity) variables, as well as sediment texture (percentage of silt, sand and gravel), landscape characteristics (percentage of the watershed covered by forest or sugar cane) and regional variables (altitude, rainfall; details in Table S1). Most specimens were identified to genus level, bearing in mind the limited taxonomic knowledge available for Neotropical fauna. Although we used genus-level data, many studies on stream macroinvertebrates have demonstrated that general community patterns hold for different taxonomic resolutions (e.g., species, genus, and family levels: [36] and references therein). The reliability of the higher-taxa approach to detect general ecological patterns depends on how species within higher taxa respond to environmental gradients. If congeners are ecologically similar to one another, ecological patterns can be detected using genus-level resolution [37]. In general, we believe that our results would be qualitatively similar if we had utilized species-level data (see also [20], [38]).

From the full dataset, which contained 242 genera, we chose five taxa to be used as predetermined indicator groups in our analyses: chironomids (non-biting flies; 52 genera), ephemeropterans (mayflies; 26 genera), trichopterans (caddisflies; 34 genera), and coleopterans (beetles; 54 genera). For different reasons, these taxa are usually regarded as reliable indicators in biomonitoring of freshwater ecosystems [39]. The remaining taxa include mainly odonates, lepidopterans, plecopterans, other dipterans, and annelids. Although chironomids are one of the most speciose groups in any tropical aquatic environment, they also require difficult and time-consuming analysis for identification to genus level or lower. Also, there is much debate on the importance of including chironomid data in biomonitoring and conservation programs [40]. On the other hand, ephemeropterans, trichopterans, and coleopterans are believed to be good indicators of water quality, and are more easily and quickly identified [39]. Although stoneflies (Plecoptera) are also used as indicator taxa in many studies, we did not consider this group individually in our analyses because of the low number of genera (7) in our dataset. We performed our analyses with these groups individually, and also with some of them combined: EPT (ephemeropterans, plecopterans and trichopterans; 67 genera) and EPTC (ephemeropterans, plecopterans, trichopterans, coleopterans; 120 genera). These groups, especially EPT, have been used extensively in biomonitoring programs in North America, Europe and Australia [39], [41].

### Spatial Predictors

We created spatial variables following Borcard et al. [42]. This approach, formerly called Principal Coordinates of Neighbor Matrices (PCNM), is similar to other spatial eigenfunction analyses that are now called MEM (Moran’s Eigenvector Maps [43]). MEM were based on a Euclidean distance matrix between sampling sites. This distance matrix was then submitted to a Principal Coordinates Analysis, in which axes (eigenvectors) are linearly uncorrelated [44]. From the entire set of eigenvectors, we selected those associated with positive eigenvalues and with significant Moran’s *I* because they represent positive spatial autocorrelation [44]. These eigenvectors (from now on termed spatial variables) were used as explanatory variables in our analyses (see [42] for further detail). Spatial variables associated with high eigenvalues represent broad-scale patterns of relationships among sampling sites, whereas those associated with low eigenvalues represent fine-scale patterns [44]. There has been recent criticism on the use of MEM in canonical ordinations, especially regarding using them as a direct representation of dispersal limitation [45], [46]. Thus, although we estimated both pure environmental and spatial components in variation partitioning (see details below), our main intention was to use spatial variables as a way to control for inflated type I error in assessing the environmental component. That is why we used MEM and interpreted pure spatial components cautiously.

### Statistical Analysis

#### Hypothesis 1: Indicator taxa are reliable surrogates of the entire metacommunity composition

To evaluate the congruence (similarity in patterns of community composition) between predetermined indicator taxa and the entire metacommunity, we computed two Principal Coordinates Analyses (PCoA), one for the indicator taxa and another for the entire metacommunity. All PCoAs were computed using the Bray-Curtis dissimilarity as the distance measure. The configurations of the site scores on the ordination axes represent the main patterns in community composition. We then compared the ordination patterns generated by a given indicator taxa and the entire metacommunity with a Procrustes rotation analysis ([47]; see step 1 in Figure S1). In Procrustes analysis, a rotational-fit algorithm is used to minimize the sum of squared residuals between the pair of matrices under comparison [48]. The resultant statistic, called *m* ^2^, was transformed into the *r* statistic (*r* = square-root of 1-*m* ^2^) and this last statistic is a measure of the level of community congruence, indicating the strength of the match between ordinations. For this comparison, we used the first three PCoA axes, which accounted for a substantial proportion of the variation in the data (Table S2). The statistical significance of each *r* statistic was assessed by randomization tests (999 permutations [48]).

#### Hypothesis 2: Indicator taxa respond to the same factors that affect the entire metacommunity

We evaluated whether the response matrices, defined either for the metacommunity or for each of the predetermined indicator groups, were correlated similarly with environmental and spatial predictors. For this task, we also used Procrustes analysis, but instead of using site scores derived from a PCoA, we computed two-dimensional site scores that are associated with (or constrained by) “pure environmental” [E/S], and “pure spatial” [S/E] components from a partial redundancy analysis (pRDA [49]). Whereas with the PCoA we obtained the main patterns in community composition for this metacommunity, with the RDA scores we obtained the main patterns in community composition constrained by either environmental or spatial variables (step 2 in Figure S1).

A second way to measure the congruence among patterns associated with structuring processes is to examine the relative importance of environmental and spatial variables in driving variation in community composition, of either the entire metacommunity or the indicator taxa. We used variation partitioning [50], [51] to estimate and test the fractions of total variation explained purely by environmental variables, and purely by spatial variables (step 3 in Figure S1). Partial RDA is a multivariate extension of multiple linear regression with corresponding *R* ^2^ that measures the amount of variation that can be attributed exclusively to each set of explanatory variables included in a RDA model. The different resulting components are: total explained variation [E+S], environmental variation [E], spatial variation [S], environmental variation without a spatial component [E|S], and spatial variation without the environmental component [S|E] (for details see [51]). For this analysis, the response variables were the biological composition, and the explanatory groups of variables were the environmental and PCNM variables. We transformed the compositional matrices using Hellinger transformation [52] prior to analyses. The results of the variation partitioning were based on adjusted fractions of variation [51]. Significance levels were computed by randomization tests (999 permutations [49]).

#### Hypothesis 3: The performance of indicator taxa depends on the amount that they contribute to the completeness of the community data

To investigate whether the performance of a predetermined indicator taxon depends more on its intrinsic indicator ability than on the number of genera that it possesses (for instance, an indicator taxon can be regarded as a good indicator simply because its number of genera approaches the total number of genera in the entire metacommunity), we repeated the above analyses using null models. In these null models, we created 1,000 subsets by selecting a given number of genera (from 10 to 240 with intervals of 10) at random from the metacommunity (see the total number of possible combinations in Table S3). Thus, for each of the 1,000 datasets generated for each number of genera (with sites on the lines, and a given number of randomly selected genera from the genus pool in the columns), we repeated the analysis of congruence described above (i.e., PCoA followed by Procrustes analysis). Also, we compared the Procrustes *r* obtained with the analysis of a particular indicator taxon matrix (e.g., ephemeropterans with 26 genera, trichopterans with 34, and so on for the other groups) with the distribution of 1,000 *r*-values obtained with the Procrustes analysis of the random subsets with the same genus richness (Figure S1). Similarly, we used the same random subsets as response matrices in a partial RDA. Thus, we analyzed the 1,000 datasets (for each genus richness) with a partial RDA, and used the estimated fractions to create the reference distributions (one for each fraction). These analyses allowed us to test the surrogacy power and the responsiveness to environmental gradients of particular indicator taxa when compared to random subsets with the same number of genera.

The above analyses can be interpreted, in general, as follows. Although Procrustes’ *r* may be statistically significant, it may not represent the highest value of congruence that can be obtained within a community. Similarly, although a pure environmental component [E/S] may be statistically significant, indicating the importance of environmental gradients, other subsets with the same number of genera may respond more strongly than the indicator taxon to these environmental gradients. However, these analyses do not indicate that a certain predetermined indicator taxon is not able to represent the ordination patterns that are generated by the entire metacommunity, or that this group is unrelated to environmental gradients. The analyses do indicate that this group may be the best possible compared to other subsets from the metacommunity. All analyses were performed in the R-language environment [53].

## Results

**Figure 1:**
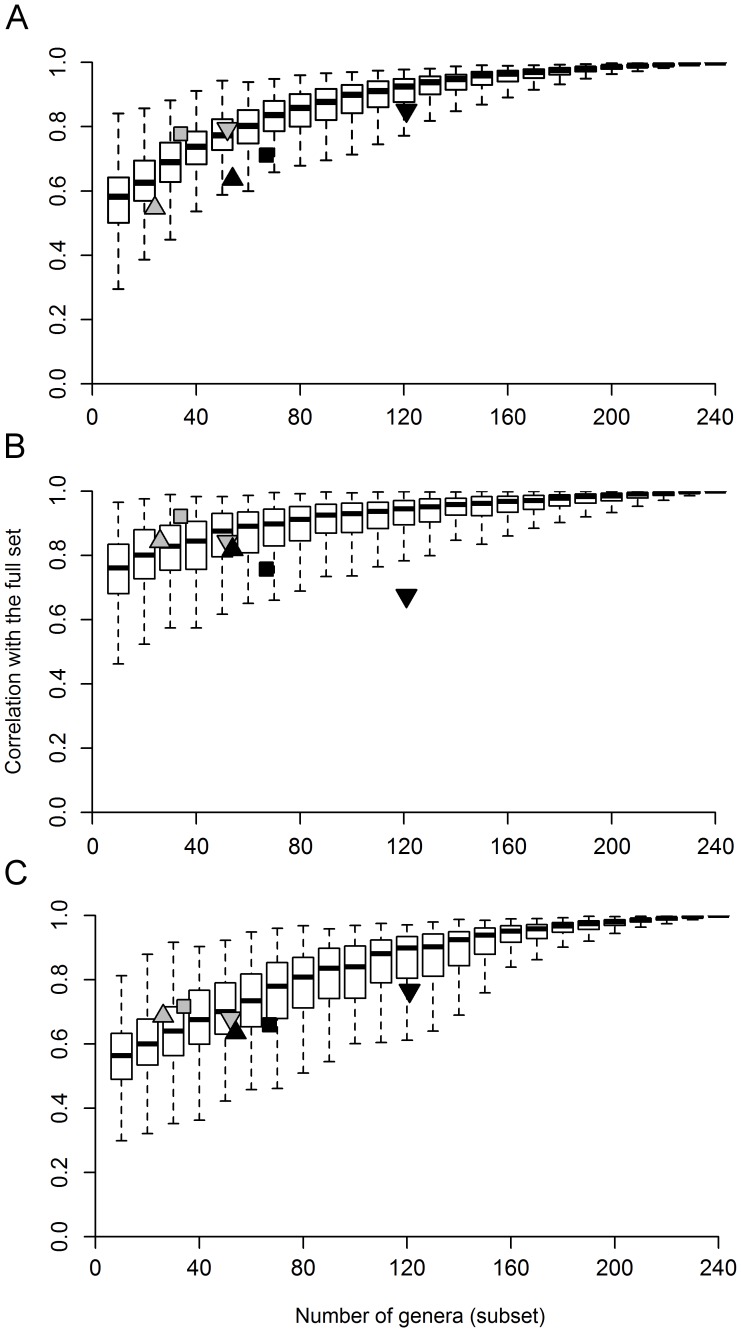
Congruence between predetermined indicator taxa and random subsets with the entire metacommunity. (A) In the main patterns in community composition; (B) Constrained by environmental variables; (C) Constrained by spatial variables. Gray triangle: ephemeropterans; gray square: trichopterans; inverted gray triangle: chironomids; black triangle: coleopterans; black square: EPT; inverted black triangle: EPTC.

**Figure 2:**
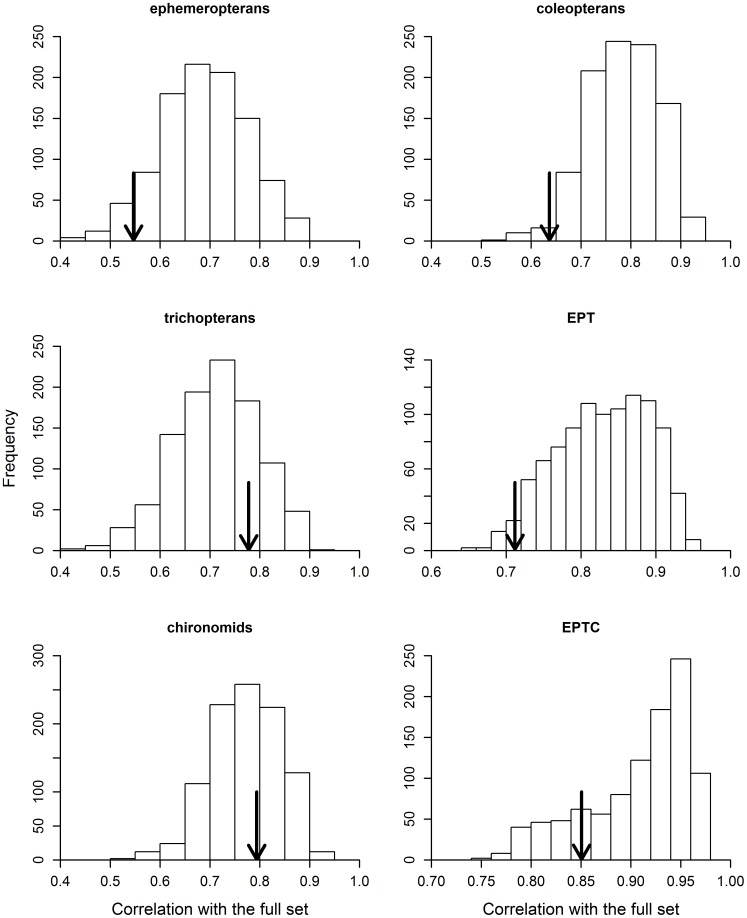
Congruence in community composition between each predetermined indicator taxon (indicated by the arrow) and between the 1,000 random subsets with the entire metacommunity. Random subsets have the same genus richness as the predetermined indicator taxon under comparison.

Some groups (e.g., chironomids) showed higher congruence in community similarity with the entire metacommunity (i.e., the full dataset) than others (e.g., ephemeropterans), but all correlations were higher than 0.5 (Figure 1A). Except for trichopterans and chironomids, most random subsets had a higher correlation with the entire metacommunity than with indicator taxa with exactly the same number of genera (Figure 2). An interesting finding here was that by using a relatively small number of genera, for example 70 genera chosen randomly from 242 (less than 1/3 of the total), in general, we would have a strong chance of reaching a correlation higher than 0.8, and in most cases higher than the congruence of the predetermined indicator taxon (Figure 1A).

**Figure 3:**
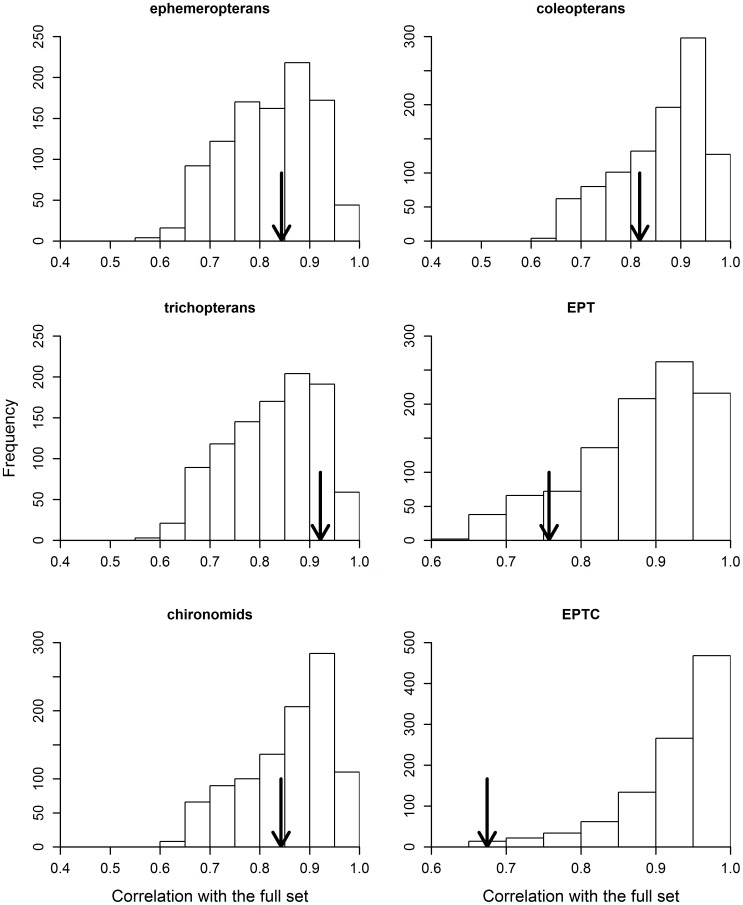
Congruence in environmentally constrained ordination axes (extracted from a pRDA) between each predetermined indicator taxon (indicated by the arrow) and between the 1,000 random subsets with the entire metacommunity. Random subsets have the same genus richness as the predetermined indicator taxon under comparison. Results regarding the congruence in spatially constrained ordination axes were very similar to that shown in this figure, and are not presented because of considerations of space.

The analysis of constrained ordination axes (resulting from pRDA) yielded similar results to those found in the previous, unconstrained analysis (Figure 1B–1C). Except for Trichoptera and Ephemeroptera, most random subsets had a higher correlation with the entire metacommunity than did the indicator taxon with an equivalent number of genera (Figure 3). In other words, the constrained ordination scores obtained with the use of random subsets were more closely correlated with the constrained ordination scores obtained with the use of the entire metacommunity as a response matrix, than with those scores derived from a particular indicator taxon.

**Figure 4:**
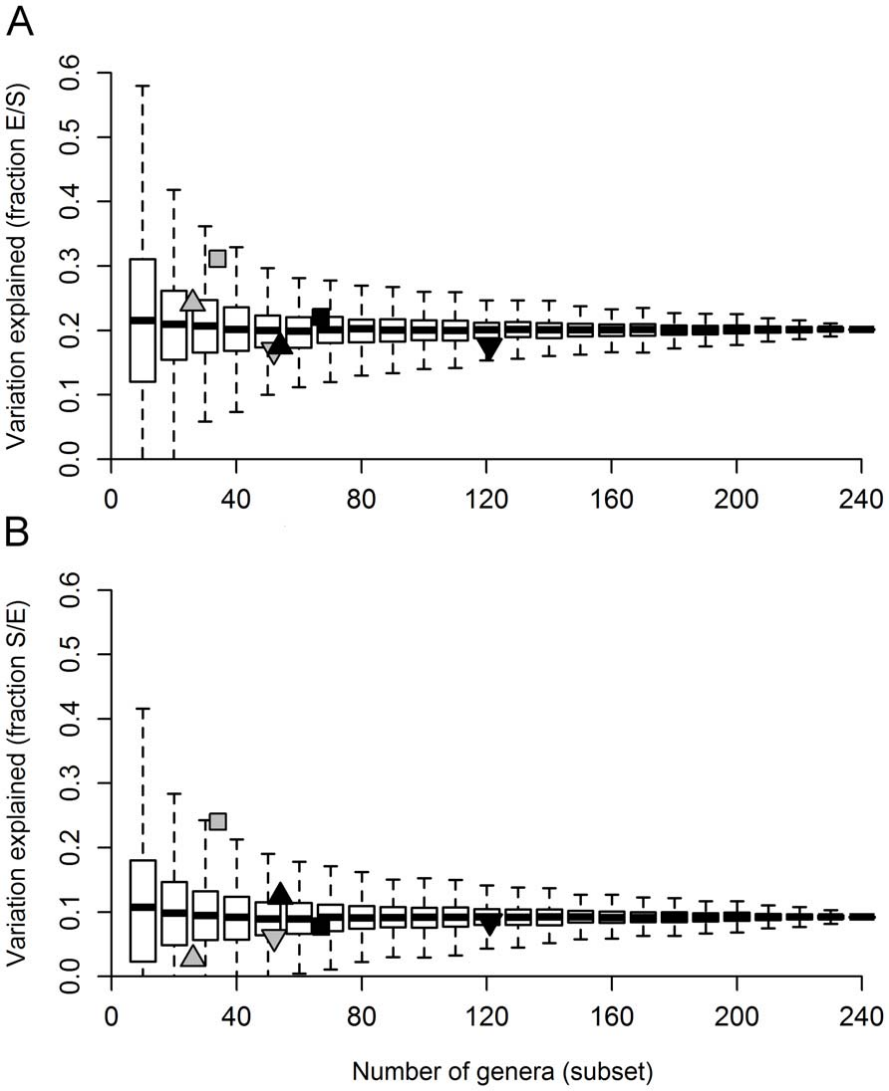
Adjusted canonical coefficients of determination associated with the “pure effects” of predictors on the predetermined indicator taxa and random subsets. (A) Pure environmental fraction; (B) Pure spatial fraction. Gray triangle: ephemeropterans; gray square: trichopterans; inverted gray triangle: chironomids; black triangle: coleopterans; black square: EPT; inverted black triangle: EPTC.

**Figure 5:**
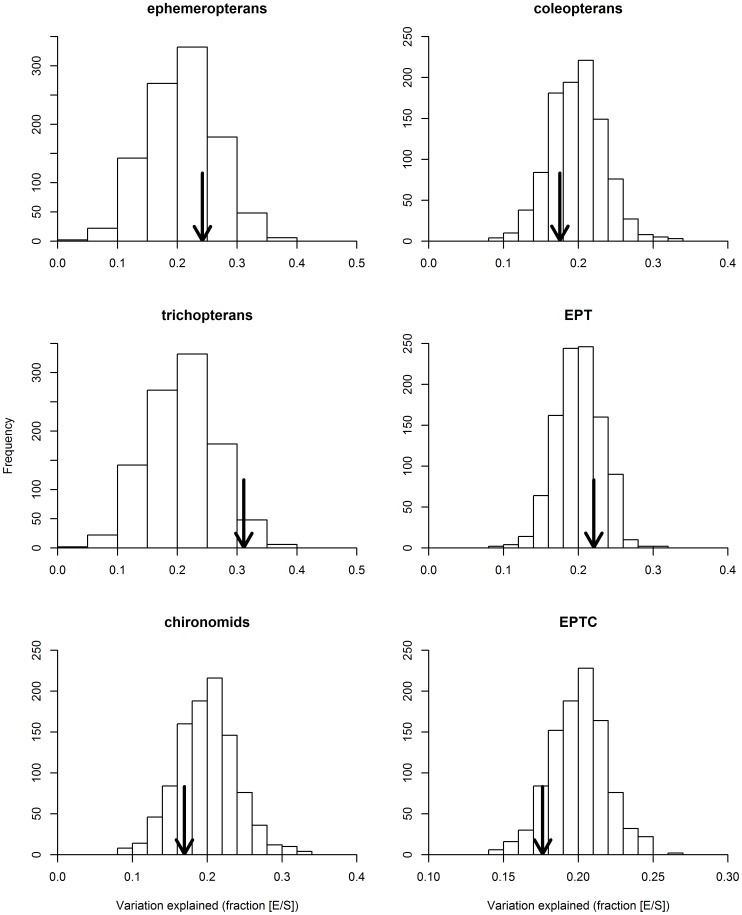
Adjusted canonical coefficients of determination associated with the “pure effects” of environmental predictors on each predetermined indicator taxon (indicated by the arrow) and random subsets. Random subsets have the same genus richness as the predetermined indicator taxon under comparison. Results regarding “pure effects” of spatial predictors were very similar to the one shown in this figure, and are not presented because of considerations of space.

Adjusted coefficients of determination (*R* ^2^_adj_) resulting from pRDA varied from 0 to almost 0.6 for the pure environmental component, and from 0 to around 0.4 for the pure spatial component (Figure 4A–4B). We found the highest amounts of variation explained for trichopterans: *R* ^2^_adj_ = 0.31 for the pure environmental component [E/S] and *R* ^2^_adj_ = 0.24 for the pure spatial component [S/E]. Also, Trichoptera had a higher correlation with the entire metacommunity than most random sets with an equivalent number of genera (Figure 5). Two general patterns emerged when we used the random subsets as response matrices in variation partitioning. First, the average amount of variation explained (ca. 20% for [E/S] and 10% for [S/E]) was unrelated to the number of genera, and similar to that obtained for the metacommunity as a whole (Figure 4A–4B). Second, for random subsets with fewer genera, especially 10 (4.12% of the total number of genera), we found the highest amount of variation explained, but the results were also more variable.

Considering this result, we decided to scrutinize in detail the 1,000 random subsets composed of 10 genera (first boxplot in Figure 4A). We found that in 340 random subsets (of 1,000), the variation in community composition was not significantly explained by the pure environmental component [E/S]. We also found that in 78 subsets (of 1,000), the amount of variation in community composition significantly explained by the pure environmental component [E/S] was higher than 40% (ranging from 40 to 58%). These subsets are potentially the best ones for use as indicators (hereafter called species sorting sets), as their composition varied widely according to the environmental gradients.

What aspects make those 78 random subsets good indicators? Was it because of the presence of certain genera, from one of the predetermined taxonomic groups? To answer these questions, we used a Kruskal-Wallis analysis to test whether the number of times in which a given genus was classified as belonging to species sorting sets depended on the taxonomic group (chironomids, ephemeropterans, plecopterans, trichopterans, coleopterans, or others). We found that whether a subset could be characterized as a species sorting set did not depend on the taxonomic group (Kruskal-Wallis’ *H* = 2.33; *P* = 0.802). The use of different subsets of taxa from the metacommunity inevitably altered the number of local communities used in the analyses. However, we found no relationship between the number of sites (of subsets composed of the fewest genera) and the adjusted *R* ^2^ values (*r* = −0.007; *P* = 0.879). Therefore, we believe that our results were robust for the spatial structures of local communities used in the analyses. Finally, we also evaluated whether patterns of commonness and rareness influenced these results, by inspecting the rank-abundance plots of, for example, 20 species sorting sets (Figure S2). We verified that these subsets well represented the general pattern found elsewhere, where many taxa were rare and few taxa were common. These results indicate, first, that higher taxonomic groups that are usually used as ecological indicators did not predominate in any of the subsets (as indicated by the Kruskal-Wallis test); and second, our inferences are not biased toward common taxa.

## Discussion

Due to severe human-induced impacts, ideally, all existing species in these environments should be regarded as targets for conservation and monitoring actions. The Brazilian Atlantic Forest is one of the most emblematic examples of this challenge, as this biome ranks among the top five biodiversity hotspots in the world. Taking our dataset as an example, if there were no financial, practical or personal constraints, we could recommend to decision-makers that all the 242 macroinvertebrates that we analyzed here should be monitored across these streams. However, this is not feasible because of the shortage of time, money, and personnel with taxonomic skills. Opportunely, our results indicate that highly diverse groups can be monitored using a few selected groups. A relatively small subset (a number between 1/4 and 1/3 of the total) would represent around 80% of the total variation in metacommunity composition. By using this subset, we would also obtain similar environmental and spatial models to those obtained by using the entire metacommunity. Surprisingly, this subset does not have to be composed of certain predetermined (in general, taxonomically defined) indicator taxa; on the contrary, it could be defined with an intensive computational search. Moreover, we show that certain random subsets composed of even fewer genera (around 5% of the total richness) could perform much better in responding to environmental (species sorting sets) and spatial gradients than the indicator taxa.

The number of taxa is expected to influence the effectiveness of indicator groups [28]. In order to avoid analytical artifacts when selecting bioindicators, it is important to evaluate the performance of indicator groups by taking the number of taxa into account. For example, except for Trichoptera, all commonly used indicator taxa showed levels of concordance with the entire metacommunity that were lower than or similar to (chironomids) random subsets, after controlling for the effect of genus richness. The performance of indicator groups will depend on the patterns of ecological complementarity between species. Therefore, groups composed of taxa that differ in their ecological requirements are expected to perform better than others. A high performance of Trichoptera can be explained by its restricted ecological niches in terms of feeding types [54] and adaptations to environmental gradients [55]. The group has been suggested to reflect the intensity of different stressors on aquatic ecosystems, and has been used as indicators in many biomonitoring programs around the world [54]. Moreover, trichopterans have other features that make them reliable biological indicators (good implementation characteristics). For example, the taxonomy of tropical Trichoptera is relatively well resolved (Trichoptera Checklist Coordinating Committee: Trichoptera World-Checklist; http://entweb.clemson.edu/database/trichopt/), and a relatively high number of trichopteran species is likely to be present per stream [56].

The responses of the entire metacommunity, indicator taxa, and random subsets to environmental and spatial gradients were partially similar to the results discussed above. Random subsets performed better in representing the constrained ordinations of the entire metacommunity than did the indicator taxa with similar numbers of genera. This was unexpected, because EPTC includes taxa that are believed to be good indicators of water quality, and are extensively used in biomonitoring programs in North America, Europe and Australia [39], [41]. These findings reinforce our view that it is the combination of certain taxa, independent of their taxonomic group, which makes a good indicator group. An ideal indicator group for environmental monitoring should have the potential to discriminate human impacts from different levels of natural variability. It is unlikely that any given taxonomic group will satisfy all these requirements in different threat scenarios. For instance, the streams that we investigated are impacted by conversion of the natural habitat for different uses, such as *Eucalyptus* and sugar-cane plantations and cattle ranching. Because close relatives tend to be ecologically similar [57] and because we were dealing with a broad taxonomic representation, as we increased the number of genera in a random subset, we also increased the probability of including genera from different taxonomic groups, with different environmental requirements and, therefore, more responsive to different environmental gradients. These random subsets with a larger number of less closely related genera would also be the most complementary subsets, showing the highest levels of concordance with the entire metacommunity. Future studies should investigate whether high concordance between the entire metacommunity and random subsets also appear in datasets with a narrow taxonomic representation.

Understanding the response of biodiversity to environmental and spatial gradients is fundamental for planning sound biological monitoring programs and for the establishment of protected areas. We showed that more than 30% of the variation in community composition of trichopterans was explained by environmental factors and 24% by spatial variables; whereas for the entire metacommunity and other indicator taxa, these values were around 20% and 10% respectively. Although, on the one hand, these findings only reinforce the view that both deterministic and stochastic processes drive variation in community composition [25], on the other hand, these findings suggest the possibility of using groups of taxa that better respond to these processes for monitoring and conservation purposes. The analysis of the random subsets composed of 10 genera showed that some subsets had a pure environmental component close to 60% (species sorting sets), whereas others showed no response to the environmental gradient. The theoretical scope that underpins the use of indicators was derived from a deterministic view of ecology, particularly based on the niche concept. Among current metacommunity frameworks, the species sorting model represents this deterministic view, in which metacommunity structure is determined by species’ responses to environmental factors; whereas the neutral model represents the other extreme, in which metacommunity structure is mainly determined by dispersal limitation, speciation and ecological drift, rather than by ecological differences among species [20]. Integrating these ideas into the scope of environmental monitoring, we suggest that in a continuum between environmental and spatial processes, the closer to the environmental extreme, the better the indicator. However, our approach can be refined further by searching for taxa – within the species sorting sets – that have specific relationships with one or another environmental variable, as this search can be informative when one is interested in selecting indicators for a particular impact. At the moment, it is important to emphasize that these subsets are composed of both common and rare taxa, and that there is no predominance of any particular higher taxon.

On the other hand, the message becomes less clear when we move to a discussion about indicators and spatial variables. Although the recognition of dispersal limitation as a fundamental process in structuring metacommunities has contributed to a better understanding of biodiversity patterns [25] and species extinctions after habitat loss [58], there is still limited discussion about the implications of this process for management, conservation and biomonitoring [26], [59]. Moreover, the only available method to include space in canonical ordinations (Moran’s Eigenvector Maps – MEM [43]), either as a way to understand spatial related processes or as a way to filter out spatial variation, has been the focus of recent criticism [45], [46]. We suggest three implications that need careful investigation, bearing in mind the current limitations of MEM. First, if the random subsets that did not respond to the environmental gradient are mainly affected by dispersal limitation, then they may be very susceptible to the spatial configuration of habitat patches (spatial component *per se* [26]). In that case, these sets would provide a powerful indication that, although different parts of the landscape are environmentally equivalent, due to historical, regional, or random processes, they support unique community compositions, and this uniqueness in itself could be a reason for conservation. Second, when one is interested in selecting indicators of habitat conditions, then monitoring these subsets (i.e., those unrelated to environmental gradients) is unnecessary, as they only introduce noise into the analysis of community-environment relationships. Although we cannot exclude the possibility that the lack of relationship between these groups and the environmental gradient may simply reflect the fact that some environmental variables are missing, from our experience in working with Atlantic Forest streams [11], [24], [29], [30], [33] and based on reviews on the subject [34], [35], most of the important environmental variables were measured. Third, spatial processes can further negatively affect the performance of indicators. For example, intense dispersal (i.e., mass effects) can mask the influence of environmental factors on species distribution (e.g., [60]). The mass-effects paradigm assumes that frequent dispersal from a source habitat enhances the persistence of a species in a sink habitat from which it would otherwise be absent [20]. In short, although mass effects are mainly documented in experimental systems (but see [61]), their occurrence could lead to inaccurate use of indicator taxa in a biomonitoring program.

Previous attempts to use subsamples in biomonitoring were based on counting a minimum number of specimens [39] – a laboratory procedure in which one counts and identifies only a random subsample taken from the entire sample during the sorting process. Our method focuses on a random subset of taxa taken from the entire metacommunity. Thus, all genera had the same chance of being chosen. Although it could be initially time-consuming, because it involves the identification of the entire metacommunity before establishing the best subsets, it has the advantage of avoiding phylogenetic autocorrelation and capturing complex information about variation in community composition (i.e., beta-diversity). The numbers that we found in our study –1/4 of the entire metacommunity for biodiversity surrogacy and random subsets of 10 genera for environmental assessment – are not cutoff points for any biomonitoring program. Each program should run its own analysis, because the output will be dependent on the regional pool. Thus, to apply the strategy that we are proposing, one should first perform a comprehensive biological survey of the region of interest. Second, after running the protocol described above, one can select the subset of taxa that best fulfills one of the objectives targeted in this paper (i.e., subsets representing ordination patterns depicted by the entire metacommunity or responding to major environmental gradients). Setting clear objectives is a fundamental step in the development of an effective monitoring program [62]. For instance, let us suppose that a high level of community congruence is required (i.e., the relationship between an indicator group and the entire metacommunity should be close to 1.0). According to our protocol, one should select approximately 120 genera, and because different combinations of 120 genera are possible, one can select, for surrogacy purposes, the combination (i.e., a genera list) that maximizes the match with the entire metacommunity. Interestingly, our approach offers flexibility in terms of choosing the best subset, because different combinations of taxa might be similarly effective in representing the entire community. We must emphasize that the use of our protocol, besides the inevitable work of sorting and counting samples, comes with the extra (computational) cost of searching for the best subsets. We envisage that in the long term this cost can be rewarding, given the small amount of time and expertise needed to analyze the samples. We advise, however, that from time to time a new complete evaluation should be carried out to assess the effectiveness of a particular subset, considering that as new data become available the goals of monitoring programs might change [62]. Thus, in terms of rationality and implementation, our approach seems to be adequate to accomplish the purpose of selecting bioindicators – it can be considered an effective method. However, studies of cost-effectiveness and cost-efficiency are necessary to know whether it performs in the best possible way and with acceptable financial and personnel costs in comparison with other approaches [63]. In addition, it would be highly desired, especially considering the transferability of our approach, if we could perform a temporal verification of the whole procedure using the same landscape.

In conclusion, the approach that we propose here, exemplified by macroinvertebrates in Atlantic Forest streams, places in the same theoretical and practical framework the selection of surrogates of biodiversity, indicators of environmental conditions, and, more importantly, it explicitly incorporates environmental and spatial processes into the selection approach. It recognizes that both the existence and lack of community-environment relationships, and relationships with spatial variables are relevant because they provide different information about the phenomenon of interest. Also, our work adds to the growing efforts [25] to apply the theoretical foundations of the metacommunity perspective.

## Supporting Information

Figure S1 **Diagram showing the step-by-step statistical methodology.** Step 1: **M** represents the entire metacommunity matrix, with all 242 macroinvertebrate genera; **I** represents a matrix composed of predetermined indicator taxa: chironomids, ephemeropterans, trichopterans, coleopterans, EPT or EPTC; **R** represents a matrix of genera randomly selected from **M**. **B** represents a matrix computed using the Bray-Curtis dissimilarity as the distance measure for each of the previous matrices **B_M_**, **B_I_**, **B_R_**. PCoA: Principal Coordinates Analysis. Step 2: **E** represents a matrix of environmental predictors; RDA: redundancy analysis. Step 3: **S** represents a matrix of spatial predictors; Variation components: [a] unique fraction of variation explained by environmental predictors, [c] unique fraction of variation explained by spatial predictors, [b] the common fraction of variation shared by environmental and spatial predictors, [d] the residual fraction of variation.
(PDF)

Figure S2 **Rank-abundance plot for 20 of the 78 random subsets with the highest** ***R*** **^2^_adj_ values of the pure environmental component.**
(PDF)

Table S1 **Summarized description of the dataset analyzed.**
(XLSX)

Table S2 **Proportion of the variation in the data explained by PCoA.**
(XLSX)

Table S3 **Total number of possible combinations of random genera.**
(XLSX)
