# Supplementary figures and images for: A Metacommunity Framework for Enhancing the Effectiveness of Biological Monitoring Strategies
Source: PLoS One. 2012 Aug 24;7(8):e43626. doi: 10.1371/journal.pone.0043626 (PMC3427378; doi:10.1371/journal.pone.0043626)

# STEP 1

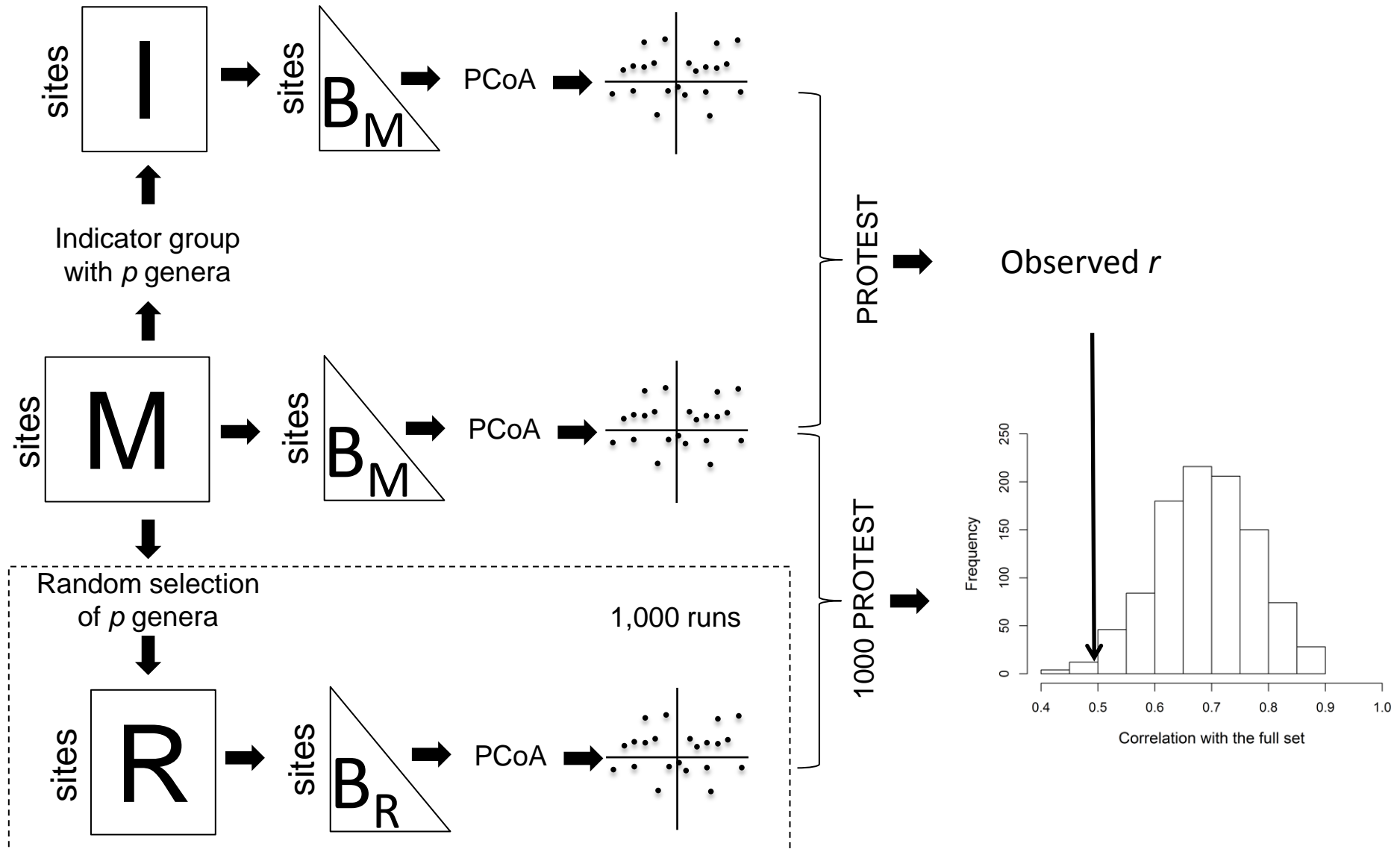

# STEP 2

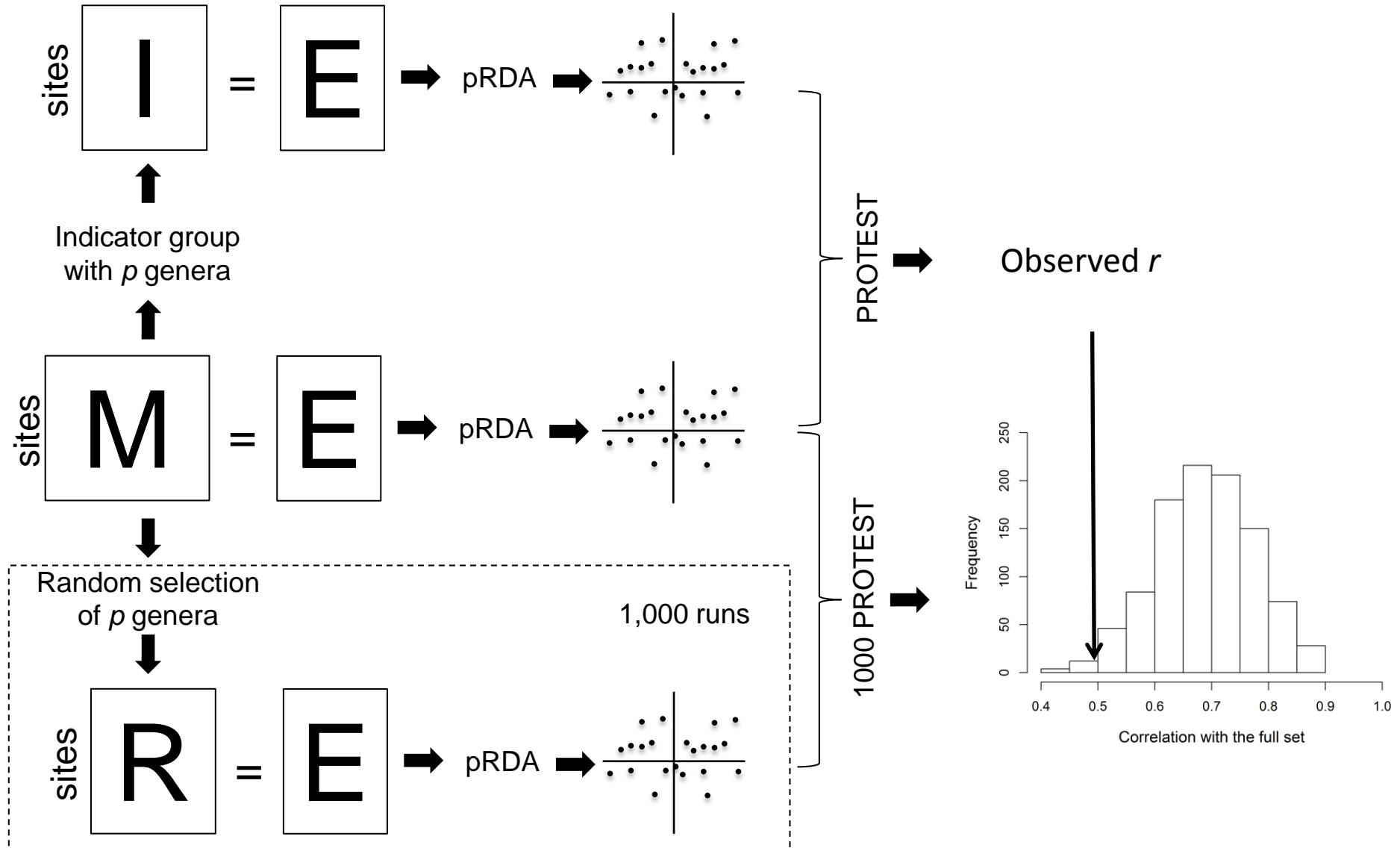

# STEP 3

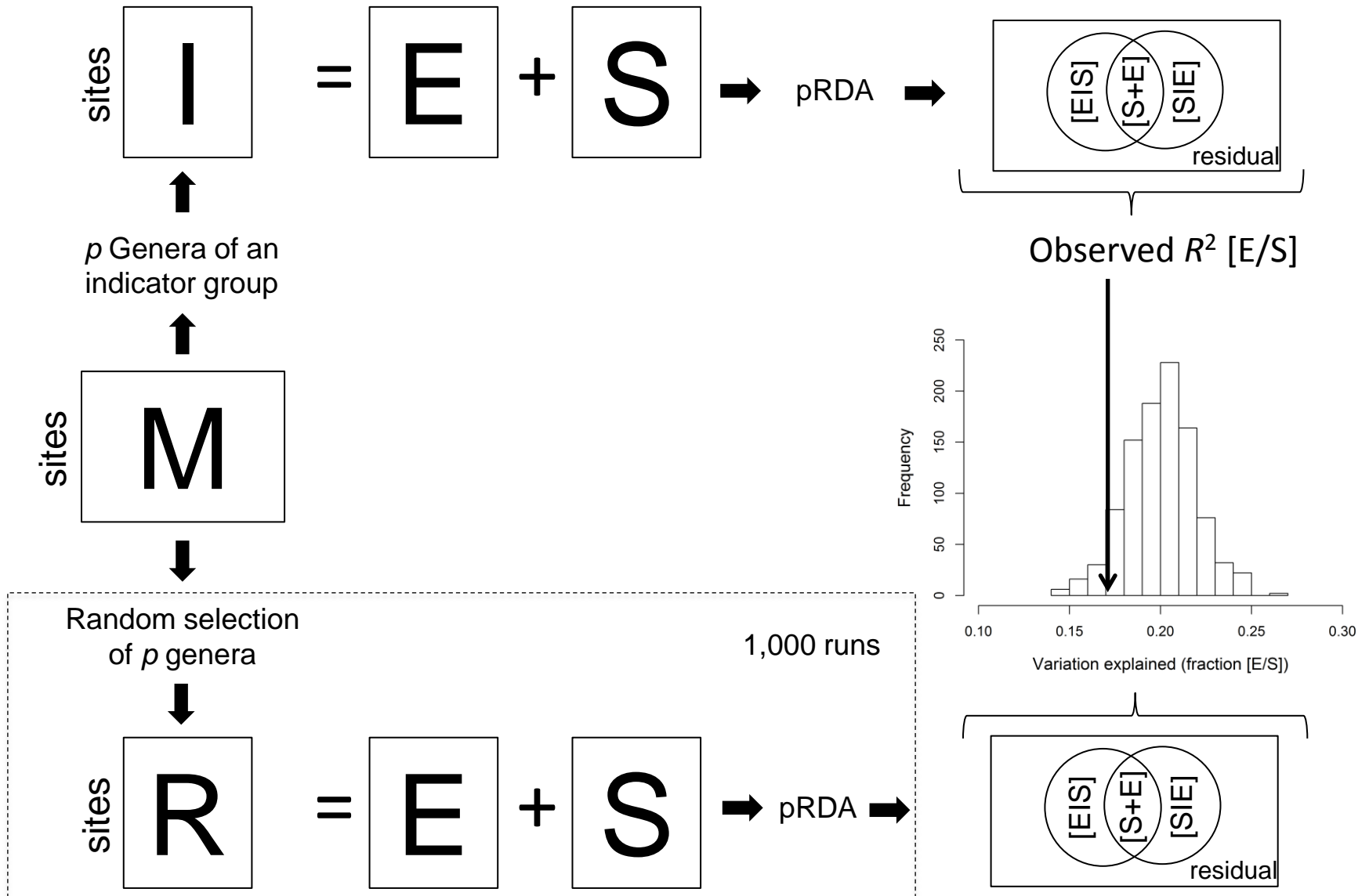

Supplement: Figure S1 — Diagram showing the step-by-step statistical methodology. Step 1: M represents the entire metacommunity matrix, with all 242 macroinvertebrate genera; I represents a matrix composed of predetermined indicator taxa: chironomids, ephemeropterans, trichopterans, coleopterans, EPT or EPTC; R represents a matrix of genera randomly selected from M. B represents a matrix computed using the Bray-Curtis dissimilarity as the distance measure for each of the previous matrices BM, BI, BR. PCoA: Principal Coordinates Analysis. Step 2: E represents a matrix of environmental predictors; RDA: redundancy analysis. Step 3: S represents a matrix of spatial predictors; Variation components: [a] unique fraction of variation explained by environmental predictors, [c] unique fraction of variation explained by spatial predictors, [b] the common fraction of variation shared by environmental and spatial predictors, [d] the residual fraction of variation. (PDF) [file pone.0043626.s001.pdf]

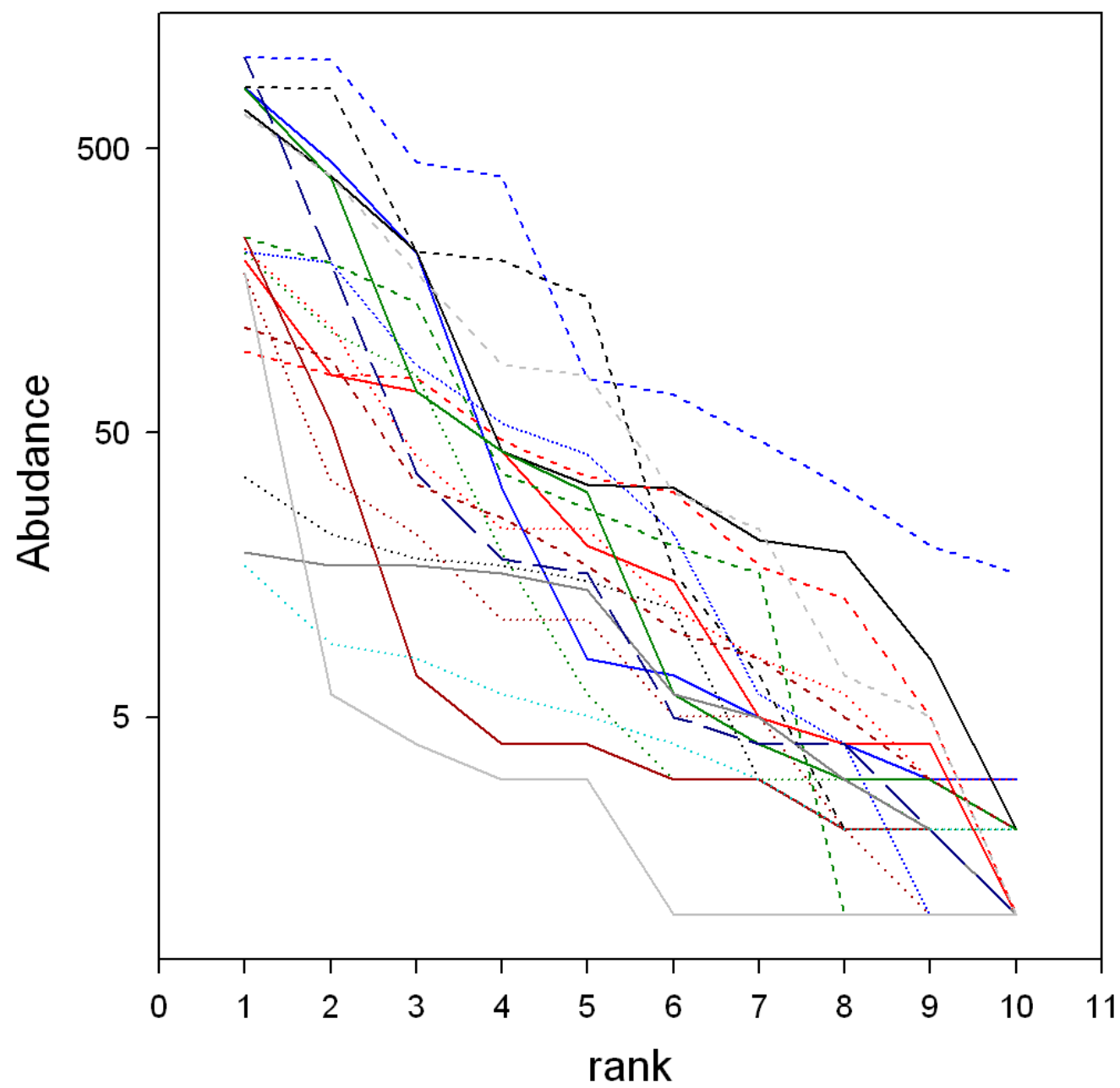

Supplement: Figure S2 — Rank-abundance plot for 20 of the 78 random subsets with the highest R 2adj values of the pure environmental component. (PDF) [file pone.0043626.s002.pdf]
